# Empirical use of fluoroquinolones improves the survival of critically ill patients with tuberculosis mimicking severe pneumonia

**DOI:** 10.1186/cc11839

**Published:** 2012-10-25

**Authors:** Yu-Tzu Tseng, Yu-Chung Chuang, Chin-Chung Shu, Chien-Ching Hung, Chiung-Fang Hsu, Jann-Yuan Wang

**Affiliations:** 1 Department of Traumatology, National Taiwan University Hospital and National Taiwan University College of Medicine, No. 7, Chung Shan S. Road, Zhongzheng District, Taipei City 10002, Taiwan; 2 Department of Internal Medicine, National Taiwan University Hospital and National Taiwan University College of Medicine, No. 7, Chung Shan S. Road, Zhongzheng District, Taipei City 10002, Taiwan

## Abstract

**Introduction:**

Empirical use of fluoroquinolones may delay the initiation of appropriate therapy for tuberculosis (TB). This study aimed to evaluate the impact of empirical fluoroquinolone use on the survival of patients with pulmonary TB that mimicked severe community-acquired pneumonia (CAP) requiring intensive care.

**Methods:**

Patients aged >18 years with culture-confirmed pulmonary TB who presented as severe CAP and were admitted to the ICU were divided into fluoroquinolone (FQ) and nonfluoroquinolone (non-FQ) groups based on the type of empirical antibiotics used. Those patients with previous anti-TB treatment or those who died within 3 days of hospitalization were excluded. The primary end point was 100-day survival.

**Results:**

Of the 77 patients identified, 43 (56%) were in the FQ group and 34 (44%) were in the non-FQ group. The two groups had no statistically significant difference in co-morbidities (95% vs. 97%, *P *> 0.99) and Acute Physiology and Chronic Health Evaluation (APACHE) II scores (21.2 ± 7.1 vs. 22.5 ± 7.5, *P *= 0.46) on ICU admission. Overall, 91% and 82% of patients in the FQ and non-FQ groups, respectively, had sputum examinations for TB within 1 week of admission (*P *= 0.46), and results were positive in 7% and 15% (*P *= 0.47), respectively. For both groups, 29% received appropriate anti-TB therapy within 2 weeks after ICU admission. The 100-day mortality rate was 40% and 68% for the FQ and non-FQ groups, respectively (*P *= 0.02). By Cox regression analysis, APACHE score <20, no bacteremia during the ICU stay, and empirical fluoroquinolone use were independently associated with survival.

**Conclusion:**

Empirical use of fluoroquinolones may improve the survival of ICU patients admitted for pulmonary TB mimicking severe CAP.

## Introduction

Severe community-acquired pneumonia (CAP), defined as pneumonia acquired in the community area that rapidly progresses to require ICU admission, is a major infectious cause of hospitalization and mortality [1]. In patients presenting with severe CAP, fluoroquinolones (FQs) have been recommended as first-line empiric antibiotic therapy due to their broad-spectrum antimicrobial effect [2]. The use of FQs has been proven to reduce the length of hospital stay and is more cost-effective than using the combination therapy of β-lactams plus macrolides [3,4].

In endemic areas of tuberculosis (TB), the clinical manifestations of pulmonary TB are highly variable and may even mimic CAP [5,6]. Although FQs have excellent *in vitro *and *in vivo *bactericidal activity against *Mycobacterium tuberculosis *[7-9], empirical use of FQ monotherapy for CAP has raised concerns regarding delays in the initiation of appropriate anti-TB therapy, an increase in mortality and the emergence of drug resistance [10-13]. However, several other studies do not corroborate these findings [14-18]. In different TB endemic areas, it is difficult to define the relationship between the duration of FQ exposure and the development of resistance to FQ. Addressing the effects of different FQs on delays or resistance is also difficult, which may explain the contradictory results.

In patients with pulmonary TB requiring intensive care, the mortality rate approaches 50% [19]. Among the deaths, about 50% occur within 26 days and 75% within 75 days after ICU admission [20]. Previous studies have demonstrated that the survival of patients with TB can be significantly improved if anti-TB therapy is started within 14 days of hospitalization [11,21,22]. Whether empirical use of FQ in critically ill patients can improve survival or can cause delays in the diagnosis of TB and increase mortality remains unclear.

This retrospective study aimed to investigate the impact of empirical FQ use on the survival of patients with pulmonary TB manifesting as severe CAP requiring intensive care, in a TB endemic area.

## Materials and methods

### Study subjects

This retrospective study was conducted at the National Taiwan University Hospital, a tertiary-care referral center in Taiwan, where the 2008 incidence and mortality rate of TB was 62 and 3.3 per 100,000 population, respectively [23]. The database of the mycobacteriology laboratory and ICU records was searched to identify TB patients between January 2005 and December 2010. The inclusion criteria were age ³18 years, culture-confirmed pulmonary TB, radiographic findings suggestive of severe CAP that rapidly progressed and required intensive care within 1 week of hospitalization, and no previous anti-TB therapy except FQs prior to ICU admission.

CAP was defined as pneumonia that developed outside the hospital setting, with classic symptoms of fever, cough and dyspnea, laboratory findings of leukocytosis, leucopenia or elevated serum C-reactive protein, and radiographic findings of pulmonary consolidation. The first-line anti-TB agents included isoniazid, rifampin, ethambutol, pyrazinamide, and streptomycin. Acid-fast smears and mycobacterial cultures of sputum and other respiratory specimens were performed as described previously [24]. Indications for ICU admission included respiratory failure or septic shock.

The identified patients were divided into two groups: patients who received empiric FQ therapy (that is, levofloxacin, moxifloxacin, and ciprofloxacin) before pulmonary TB was confirmed by culture (FQ group); and those who received antibiotics other than FQs (non-FQ group). The hospital's Research Ethics Committee approved the study (NCT registration number 9561707008). Informed consent was waived.

### Data collection

All of the medical records for the enrolled patients were reviewed. Respiratory symptoms were defined as cough, dyspnea, and hemoptysis. Gastrointestinal symptoms were defined as nausea, vomiting, gastrointestinal bleeding, or diarrhea. Steroid use was defined as use of >10 mg prednisolone daily during hospitalization [25]. Chest images were independently reviewed by two pulmonologists. If a discrepancy was noted between their interpretations, the image was further reviewed by a third pulmonologist who was blinded to the results.

Bacteremia in the ICU was defined as positive blood cultures obtained during the ICU stay, with the exclusion of contaminated culture results (for example, only one in two sets of blood cultures that reported *Bacillus *or coagulase-negative staphylococci). The Acute Physiology and Chronic Health Evaluation (APACHE) II score and the Sequential Organ Failure Assessment score within the first 24 hours of ICU admission were used to evaluate disease severity and organ dysfunction [26,27]. Patients who received immunosuppressants, anti-cancer chemotherapy, radiation, and steroids [25], or had another disease that was sufficiently advanced to suppress resistance to infection (for example, leukemia, lymphoma, or acquired immunodeficiency syndrome), were defined as immunocompromised hosts. The primary end point was 100-day survival.

### Statistical analysis

Inter-group difference was calculated using the independent-samples *t *test for continuous variables and the chi-square test or Fisher's exact test for categorical variables, as appropriate. Time-to-event curves were generated by the Kaplan-Meier method and compared using the log-rank test. Cox proportional hazards regression analysis was performed to identify prognostic factors for 100-day survival after ICU admission. The potential factors included age, sex, empirical FQ use, immunocompromised status, APACHE score (³20 vs. <20) [28], Sequential Organ Failure Assessment score (³8 vs. <8) [29], albumin level (<3 vs. ³3) [30], bacteremia during ICU stay, performance of acid-fast smears and mycobacterial cultures of respiratory specimens within 1 week of ICU admission, grading of acid-fast smears, and initiation of anti-TB therapy within 2 weeks of ICU admission. Significance levels for entry into the stepwise variable selection procedure were set at 0.15. Two-sided *P *< 0.05 was considered significant. All statistical analyses were performed using SPSS version 18.0 (SPSS Inc., Chicago, IL, USA).

## Results

Between January 2005 and December 2010, 1,928 patients with culture-confirmed pulmonary TB were identified. Among these, 184 patients (9.5%) were admitted to the ICU during their hospital stay, including 77 (4.0%) who presented as severe CAP that resulted in respiratory failure or septic shock, and were enrolled for this study. Of the 107 patients who were excluded, 61 (33.2%) were suspected of having TB and received anti-TB therapy before their ICU admission, 29 (15.8%) had no airway symptoms on presentation, 13 (7.1%) had been hospitalized for more than 1 week before the ICU admission, three (1.6%) died within 3 days after the ICU admission, and one (0.5%) had no sufficient medical information.

Of the 77 patients presenting severe CAP, empirical FQs were given to 43 patients before the date of diagnosis of culture-confirmed pulmonary TB (FQ group), including 30 patients who received levofloxacin, seven ciprofloxacin, and seven moxifloxacin (one patient received more than one FQ during hospitalization). All of these 43 patients took FQs for more than 3 days (range, 3 to 155 days; mean duration FQ intake, 9.5 days). Of the seven patients who received ciprofloxacin, the FQ was started when a diagnosis of nosocomial infection was made more than 3 days after admission. All patients in the FQ and non-FQ groups received other β-lactam antibiotics before their ICU stay, including amoxicillin/clavulanic acid in 11.6% and 14.7% (*P *= 0.74), respectively, ampicillin/sulbactam in 20.9% and 35.3% (*P *= 0.20), respectively, anti-pseudomonal antibiotics in 53.5% and 61.8% (*P *= 0.50), respectively, and linezolid in 2.3% and 0% (*P *> 0.99), respectively.

All except one patient (98%) in the FQ group had undergone investigations for other respiratory pathogens, which included urine antigen tests for Legionella, serology tests for *Mycoplasma pneumoniae*, and nasal swab for influenza. One patient tested positive for influenza and another had concurrent pneumococcal pneumonia with bacteremia that caused respiratory failure. In the non-FQ group, 62% had undergone studies for other respiratory pathogens and only one tested positive for *Mycoplasma *IgM.

Based on the clinical characteristics of the FQ and non-FQ groups (Table 1), there were no statistically significant differences in age, sex, co-morbidities, APACHE score, Sequential Organ Failure Assessment score, and radiographic findings between the two groups. The HIV sero-status was available in 34 patients, including two in the non-FQ group who were sero-positive for HIV. Of the other 43 patients with unknown HIV sero-status, none had AIDS-defining opportunistic illnesses except TB during follow-up [31]. More patients in the FQ group had fever as the initial symptom than in the non-FQ group (63% vs. 35%, *P *= 0.02). Mean serum albumin levels were <3 g/dl in both groups. Five patients in each group did not need intubation on ICU admission and all five in the FQ group survived, while two patients (40%) in the non-FQ group died.

**Table 1:** Clinical characteristics of patients in the fluoroquinolone and nonfluoroquinolone groups on ICU admission

	Fluoroquinolone (*n *= 43)	Nonfluoroquinolone (*n *= 34)	*P *value
Age (years)	72.9 ± 12.4	76.2 ± 14.5	0.28
Male	33 (77)	26 (77)	0.99
Underlying diseases	41 (95)	33 (97)	>0.99
Diabetes mellitus	12 (28)	6 (18)	0.29
COPD	5 (12)	9 (27)	0.09
Malignancy	8 (19)	5 (15)	0.65
Autoimmune disease	2 (5)	1 (3)	0.7
Liver cirrhosis	2 (5)	0 (0)	
HIV	0 (0)	2 (6)	
Initial symptoms			
Respiratory symptoms	40 (93)	32 (94)	>0.99
Fever	27 (63)	12 (35)	0.02
Consciousness disturbance	11 (26)	4 (12)	0.13
APACHE score	21.2 ± 7.1	22.5 ± 7.5	0.46
Arterial pH	7.4 ± 0.1	7.4 ± 0.1	0.19
Serum sodium	136.6 ± 6.7	135.0 ± 8.5	0.35
Serum potassium	3.9 ± 0.8	4.1 ± 0.7	0.26
Serum creatinine	1.8 ± 1.7	1.9 ± 1.8	0.87
Hematocrit	32.5 ± 6.8	32.6 ± 5.6	0.93
Leukocyte count (10^3^/μl)	13.9 ± 8.5	12.8 ± 7.2	0.53
FiO_2 _³50%	32 (74)	20 (59)	0.22
Albumin (g/dl)	2.7 ± 0.5	2.8 ± 0.5	0.29
Bacteremia in ICU^a^	5 (12)	6 (18)	>0.99
SOFA score	8.0 ± 3.6	7.3 ± 3.9	0.47
Radiographic findings			
Bilateral involvement	34 (79)	27 (79)	>0.99
Cavitation	3 (7)	0 (0)	0.25
Miliary	1 (2)	1 (3)	>0.99
Pleural effusion	21 (49)	21 (62)	0.36
Smear-positive for acid-fast bacilli	3 (7)	5 (15)	0.47
Steroid use	21 (49)	19 (56)	0.65
Undergone bronchoscopy	5 (12)	5 (15)	0.95

Data presented as *n *(%) or mean ± standard deviation. APACHE, Acute Physiology and Chronic Health Evaluation score; COPD, chronic obstructive pulmonary disease; FiO_2_, the fraction of inspired oxygen; SOFA, Sequential Organ Failure Assessment score. ^a^Blood culture was positive for *Pseudomonas aeruginosa *in two patients, *Acinetobacter baumannii *in two patients, and for *Proteus *spp., *Enterobacter *spp., *Escherichia coli, Klebsiella pneumoniae, Streptococcus pneumoniae, Staphylococcus haemolyticus, Corynebacterium *spp., *Burkholderia cepacia, Candida parapsilosis*, and *Fusobacterium nucleatum *in one patient each. Two patients had more than one pathogen.

There were no statistically significant differences between the two groups in the proportion of patients who underwent sputum TB examination within 1 week and those who started anti-TB therapy within 2 weeks of ICU admission (Table 2). However, more patients in the non-FQ group than in the FQ group died before anti-TB therapy was started (27% vs. 9%, *P *= 0.05). Among the 39 patients (91%) who received anti-TB therapy in the FQ group, 16 continued to receive FQ for >7 days together with anti-TB therapy.

**Table 2:** Treatment course and outcome in the fluoroquinolone and nonfluoroquinolone groups

	Fluoroquinolone (*n *= 43)	Nonfluoroquinolone (*n *= 34)	*P *value
ICU admission to TB study (days)	1.7 ± 5.9	5.6 ± 15.1	0.13
TB study in the ICU within 1 week	39 (91)	28 (82)	0.46
Died before ATT was begun	4 (9)	9 (27)	0.05
Initiation of ATT in the ICU within 2 weeks	11 (29)	7 (29)	>0.99
Length of ICU stay (days)	30.0 ± 20.5	17.5 ± 17.9	<0.01
Nonsurvivors in the ICU	27.0 ± 18.0	22.0 ± 25.0	0.27
Survivors in the ICU	31.0 ± 21.6	16.0 ± 14.0	0.03
Intubation period (days)	38.6 ± 61.5	19.5 ± 24.0	0.09
Nonsurvivors in the ICU	28.0 ± 18.35	25.0 ± 29.2	0.13
Survivors in the ICU	43.0 ± 71.5	16.9 ± 21.4	<0.01
Length of hospital stay(days)	71.2 ± 62.7	38.4 ± 25.5	<0.01
Nonsurvivors in the hospital	42.0 ± 28.0	37.0 ± 26.41	0.83
Survivors in the hospital	81.0 ± 63.3	40.0 ± 25.4	<0.01
100-day mortality rate	17 (40)	23 (68)	0.02

Data presented as *n *(%) or mean ± standard deviation. ATT, anti-tuberculosis treatment; TB, tuberculosis.

For the FQ and non-FQ groups, the interval between start of investigations for pulmonary TB and the start of anti-TB therapy were 20.9 ± 14.7 and 16.8 ± 18.0 days (*P *= 0.32), respectively. The interval between hospital admission to initiation of anti-TB therapy was 24.1 ± 15.6 and 24.7 ± 17.2 days (*P *= 0.89), respectively. The lengths of ICU stay (30.0 ± 20.5 days vs. 17.5 ± 17.9 days, *P *< 0.01) and hospitalization (71.2 ± 62.7 days vs. 38.4 ± 25.5 days, *P *< 0.01) were significantly longer in the FQ group than in the non-FQ group.

Anti-TB susceptibility tests for the 77 isolates of *M. tuberculosis *revealed resistance to streptomycin in two cases and one case each to ethambutol co-resistance to isoniazid and ethambutol, co-resistance to isoniazid and streptomycin, and triple-resistance toisoniazid, rifampin plus ethambutol. None of the isolates were resistant to ofloxacin. Follow-up sputum samples were collected in all of the 77 patients, and 26 (33.8%) were culture positive for *M. tuberculosis*, including 15 in the FQ group. Of the 26 isolates, anti-TB susceptibility testing was repeated in 10, including four in the FQ group. The susceptibility patterns of the 10 follow-up isolates were identical to those of their corresponding initial isolates.

Forty patients (52%) died within 100 days of ICU admission, including 17 (39.5%) in the FQ group and 23 (67.6%) in the non-FQ group. Twenty-three patients (57.5%) died during their ICU stay, while 17 (42.5%) died after their transfer out of the ICU. The cause of death was pneumonia in 26 patients (65%) and hypercapnic respiratory failure, ischemic bowel disease, acute respiratory distress syndrome with shock, sepsis with profound shock, disseminated TB confirmed by bone marrow and urine sample cultures, and multi-drug-resistant *Acinetobacter baumannii *empyema in one patient each. Another eight patients died after hospital discharge. Their deaths were judged to be attributed to TB by the Taiwan Center for Disease Control.

**Figure 1:**
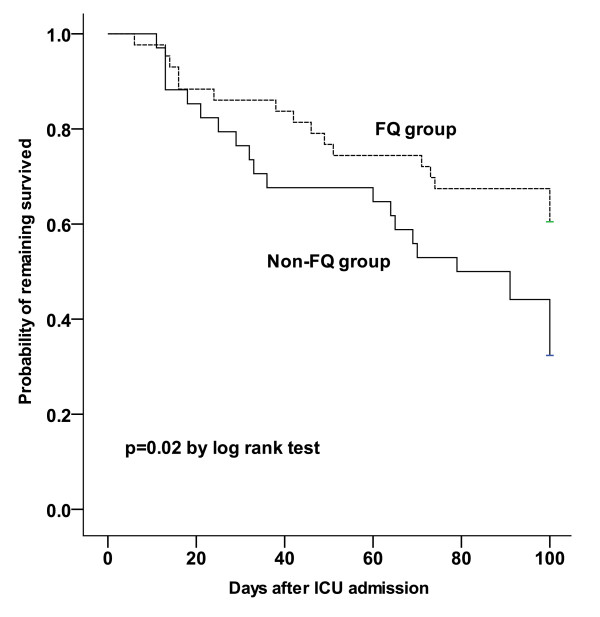
**Kaplan-Meier 100-day survival curves for patients in the fluoroquinolone and non-fluoroquinolone groups**. The fluoroquinolone (FQ) and non-FQ groups were compared using the log-rank test.

The survival patterns of patients in the FQ and non-FQ groups are shown in Figure 1. By Cox regression analysis, APACHE score ³20 (hazards ratio = 3.75; 95% confidence interval = 1.34 to 10.44), bacteremia in the ICU (hazards ratio = 3.88; 95% confidence interval = 1.64 to 9.19), and empiric use of FQ (hazards ratio = 0.36; 95% confidence interval = 0.17 to 0.77) were independently associated with 100-day survival (Table 3).

**Table 3:** Independent factors associated with 100-day survival, by multivariate Cox proportional hazards regression analysis

	Patients (*n*)	Mortality within 100 days, *n *(%)	Median survival of fatal cases (days)	*P *value	Hazards ratio	95% confidence interval
Age						
≥70	60	34 (56.7)	50	0.32	1.75	0.58 to 5.31
<70	17	6 (35.3)	16			
APACHE score						
≥20	52	33 (63.5)	36	0.01	3.75	1.34 to 10.44
<20	25	7 (28.0)	100			
SOFA score						
≥8	35	19 (54.3)	44	0.21	1.63	0.76 to 3.49
<8	41	20 (48.8)	100			
Empiric antibiotic						
Fluoroquinolone	43	17 (39.5)	46	<0.01	0.36	0.17 to 0.77
Nonfluoroquinolone	34	23 (67.6)	60			
Bacteremia in the ICU						
Yes	11	9 (81.8)	46	<0.01	3.88	1.64 to 9.19
No	66	31 (47.0)	49			
TB study in the ICU within 1 week						
Yes	67	36 (53.7)	47.5	0.22	1.99	0.66 to 5.99
No	10	4 (40.0)	48.5			
Initiation of ATT in ICU within 2 weeks						
Yes	18	10 (55.6)	68.5	0.53	1.38	0.50 to 3.84
No	44	17 (38.6)	40			

APACHE, Acute Physiology and Chronic Health Evaluation score; ATT, anti-tuberculosis treatment; SOFA, Sequential Organ Failure Assessment score; TB, tuberculosis.

## Discussion

In the present study, 4% of the patients with culture-confirmed pulmonary TB in Taiwan presented with clinical and radiographic manifestations similar to severe CAP that required intensive care support. The interval between ICU admissions and start of anti-TB treatment was about 3 weeks on average. The 100-day mortality for such patients was as high as 52% (40/77). While the severity of pneumonia on presentation and bacteremia during the ICU stay had an adverse effect on survival, empirical use of FQs may improve survival.

The finding of a high mortality rate in TB patients presenting as severe CAP requiring intensive care is consistent with that of a previous study [32] - which had similar characteristics of the study population, including old age, presence of underlying diseases, malnutrition, and lack of anti-TB treatment within 14 days of the initial visit. The causes for such high mortality rates may be delays in diagnosis and in the timely initiation of appropriate anti-TB therapy. As shown in this study, most of the patients were older people, with a mean age of 72 years, and had multiple co-morbidities. Consideration of other competing bacterial etiologies for pneumonia may cause delays, on top of concerns regarding the adverse effects of empirical initiation of anti-TB therapy before conventional approaches to the diagnosis of pulmonary TB are completed to provide guidance.

FQs are broad-spectrum antimicrobial agents recommended for patients with severe CAP requiring ICU admission [2]. Despite concerns that empirical use of FQs for severe CAP may be associated with delayed diagnosis and treatment of pulmonary TB [11,33], the findings in this study suggest that empirical use of FQs may actually improve the prognosis of TB patients presenting as severe CAP that require intensive care. Aside from benefits of activity against common bacteria that cause severe CAP in older people, FQs in this special condition may also provide survival benefit by rapidly reducing the mycobacterial load [34,35]. In this study, 65% of patients in the FQ group had sputum culture conversion after FQ monotherapy. Although more prospective studies with larger sample sizes are warranted to confirm the findings here, the results suggest that the delay in diagnosis of active pulmonary TB and the timely initiation of anti-TB therapy may be prevented in TB endemic areas if investigations for pulmonary TB are routinely performed for patients with severe CAP.

β-Lactams such as amoxicillin/clavulanate may also have antibacterial activity against *M. tuberculosis *[36]. A population-based cohort study in Canada between 1997 and 2006 reported that previous treatments with any antibiotics, not only FQ, are associated with delayed TB diagnosis [37]. Use of β-lactams is probably not a confounding factor in the survival analysis, however, because all of the patients in this study received β-lactams on admission. There is no significant difference in the proportion of patients who received β-lactam antibiotics. Moreover, there is no significant difference in the intervals from the time of pulmonary TB investigation or from ICU admission to the start of anti-TB therapy between the FQ and non-FQ groups.

The findings of APACHE score ≥20 and bacteremia during the ICU stay as poor prognostic factors are consistent with those of previous studies [28,38]. Nearly 80% of patients belong to the older age group, which may preclude identifying age ≥70 years as an independent risk factor in this study. In contrast to previous reports [11,21,22], initiation of standard anti-TB therapy within 2 weeks of ICU admission has no consistent significant impact on survival. This is probably due to the small case number in this study and due to less than one-third of the patients being treated early after admission to the ICU.

This study has several limitations such that interpretation of the results should be made with caution. First, this is a retrospective study and biases and missing data are unavoidable. For example, HIV sero-status and the cause of death are unknown in 43 and eight patients in the FQ and non-FQ groups, respectively. However, this may not be such a serious concern because HIV prevalence in the general population (<0.1%) and in patients with TB (<1%) remains low in Taiwan [39]. None of the patients with unknown HIV sero-status developed AIDS-defining illness during follow-up. Second, the study was conducted on older patients with clinical presentations of severe CAP in a medical center. The results may not be applicable to all patients with pulmonary TB that do not require critical care. Third, conventional diagnostic tests for TB (acid-fast smears and culture) are used in this study and, as such, appropriate anti-TB therapy was not initiated in the patients until 3 weeks into their hospital stay. The impact of molecular technology on facilitating rapid TB diagnosis in this clinical setting warrants further studies. Fourth, the case number of pulmonary TB mimicking severe CAP remains small so the number of *M. tuberculosis *isolates submitted for drug susceptibility tests obtained during FQ treatment is also small. The risk for emergence of acquired resistance to FQs cannot thus be adequately evaluated. Lastly, despite efforts to adjust for the confounding effects of disease severity and organ dysfunction, the decision of choosing empiric therapy in the face of severe CAP may be based on other factors (for example, drug allergy, toxicity profile, and previous medication history) that are not included in the statistical model.

## Conclusion

Empirical use of FQs may improve survival of patients admitted to the ICU for pulmonary TB mimicking severe CAP.

## Key messages

• Four percent of the patients with culture-confirmed pulmonary TB in Taiwan present as severe CAP requiring intensive care.

• The mortality rate is high in TB patients presenting as severe CAP requiring intensive care.

• Empirical use of FQs may improve survival for TB patients presenting as severe CAP requiring intensive care.

## Abbreviations

APACHE: Acute Physiology and Chronic Health Evaluation; CAP: community-acquired pneumonia; FQ: fluoroquinolone; TB: tuberculosis.

## Competing interests

The authors declare that they have no competing interests.

## Authors' contributions

Y-TT reviewed the medical records, analyzed and interpreted the data, and drafted the manuscript. C-CH, Y-CC, C-CS, and C-CH, analyzed and interpreted the data, and drafted the manuscript. C-FH reviewed the medical records. J-YW designed and oversaw the study, analyzed and interpreted the data, and revised the manuscript. All authors read and approved the manuscript for publication.
